# Antimicrobial redistribution: health practitioner One health perspectives on antimicrobial use in livestock for antimicrobial resistance education in Zimbabwe

**DOI:** 10.1093/jacamr/dlag019

**Published:** 2026-02-17

**Authors:** Martin Mickelsson, Tungamirirai Simbini

**Affiliations:** Department of Earth Sciences, University of Gothenburg, Box 460, Gothenburg SE-40530, Sweden; Department of Women´s and Children´s Health, Uppsala University, Uppsala University Hospital, Uppsala SE-751 85, Sweden; Department of Biomedical Informatics and Biomedical Engineering, University of Zimbabwe, P.O. Box A178 Avondale, Harare, Zimbabwe

## Abstract

**Background and objectives:**

Antimicrobial resistance (AMR) is a growing challenge to global health, encompassing human, animal and environmental health. In Zimbabwe, AMR is driven by antimicrobial misuse, weak infection control and poor regulation of antimicrobial use in agriculture. While policy has identified education as key in addressing AMR, there are gaps in AMR education frameworks. This paper explores Zimbabwean health practitioners’ understandings of the interconnections between human, animal and environmental health practices and how these understandings can strengthen AMR education frameworks to address antimicrobial misuse.

**Methods:**

Operationalizing participatory workshops at two hospitals in Harare, the paper qualitatively analyses discussions from 25 doctors, nurses and pharmacists on the interconnections between human, animal and environmental health in AMR development and challenges of AMR educational practice.

**Results:**

The paper identifies how health practitioners perceive recurring antimicrobial redistribution pathways from patients to (i) livestock for disease prevention and growth, (ii) family and community through informal sharing and (iii) local environment through unintentional contamination. Economic pressures, lack of knowledge and awareness and weak regulatory enforcement are perceived as drivers of antimicrobial redistribution, emphasizing the importance of AMR education to engage with both behavioural and systemic drivers as part of a One health framework.

**Conclusions:**

The paper contributes to AMR research by mapping perceived drivers of antimicrobial redistribution, highlighting how One health approaches can be integrated into AMR education frameworks. A cross-sectoral educational approach is proposed for engaging health practitioners, veterinarians, farmers, communities and policymakers in antimicrobial stewardship and behavioural change for more sustainable use of antimicrobials.

## Introduction

Antimicrobial resistance (AMR) occurs when microorganisms develop resistance to drugs designed to kill them, rendering standard treatments ineffective.^1,2^ As an emerging global sustainability challenge, AMR is a risk for human, animal and environmental health, with serious implications for food security and economic stability.^3–8^ As part of recent systemic analyses of the burden of AMR, it was estimated in 2021 that 4.71 million deaths globally could be linked with AMR, of which 1.14 million were directly connected to resistant infections. Furthermore, the analysis proposes that annual deaths would continue to increase without effective ways to address AMR.^6,8^

In Zimbabwe, AMR is driven by antimicrobial misuse, weak infection control, unregulated livestock medication and environmental contamination.^9–13^ Antimicrobial access in the country includes formal pathways of clinical prescriptions and pharmacy dispensing, characterized by limited pharmaceutical stock and significant demand, and informal pathways of private pharmacies, street vendors and leftover antimicrobials.^14,15^ While formally restricted, previous research has indicated significant over-the-counter access, along with the possibility to get antimicrobials from the veterinary sector.^16,17^ Through these multiple pathways, antimicrobials come to circulate across the domains of human and animal health, enabling the repurposing, sharing and reselling of antimicrobials.

Efforts to address AMR in Zimbabwe have centred around the One health national action plan (NAP), which builds on the Global Action Plan on AMR outlined by the WHO.^18,19^ A key aspect of the NAP is the need to develop antimicrobial stewardship and address AMR not just in the healthcare system but across human, animal and environmental domains. However, policy implementation and monitoring throughout Zimbabwe are constrained by gaps. These gaps have been ascribed to limitations in regulatory capacity, available resources, including for communication and education, as well as veterinary extension services and monitoring, especially when it comes to small-scale farming. While articulating ambitious policy goals, the NAP is thus limited by economic and structural conditions, which impact antimicrobial use and how these pharmaceuticals move throughout the human, animal and environmental domains.^20–22^

A One health perspective, which recognizes the interdependence of human, animal and environmental health, is essential for tackling AMR.^23,24^ Such an approach provides a comprehensive analytical framework for understanding AMR as a multidimensional issue influenced by human behaviour, agricultural practices, and environmental contamination.^25–30^ This study operationalizes a One health framework to study how health practitioners understand the interconnections between health domains as part of recognizing the interconnected nature of health across species and ecosystems.^25–29^

Education and training are key to improving antimicrobial stewardship.^18,19,30^ Despite global and national strategies such as the WHO Global Action Plan and Zimbabwe’s One Health National Action Plan, implementation faces barriers including limited resources, rural-urban healthcare gaps and competing needs for food security.^18,19,25,31–36^ Farmers’ perceptions strongly influence antimicrobial use in animals, with economic pressures often outweighing long-term resistance concerns.^37–41^ Addressing AMR, therefore, requires moving beyond clinical settings to understand how antimicrobials circulate through farming and community practices.^15,42–47^ While the study does not investigate these practices, it focuses on how health practitioners understand, interpret and sense-make around these farming and community practices. Health practitioners occupy a key role in the prescription of antimicrobials and counselling of patients around their use, as their understandings shape how counselling, education and stewardship efforts engage with farming and community practices.

The paper identifies antimicrobial redistribution as a key theme and analytical concept to capture how participants describe the movement of antimicrobials beyond their intended clinical use across human, animal and environmental domains. These movements include processes of repurposing antimicrobials for animal use, informal sharing of antimicrobials with others, as well as unintended environmental contamination through waste and agricultural runoff. Antimicrobial redistribution as an analytical concept is studied based on the understandings and perceptions of health practitioners and does not encompass other aspects of antimicrobial misuse, diversion or leakage. While these processes are treated as analytically distinct, they are interconnected and understood by workshop participants as contributing to cross-domain antimicrobial movements and antimicrobial resistance.

While the redistribution processes differ in terms of their character, intention, as well as policy and monitoring context, health practitioners recurrently perceived them as interconnected feedback loops driving resistance. The analytical concept of antimicrobial redistribution thus acknowledges this interconnection without collapsing the distinctiveness of the processes of antimicrobial movement.

### Research objective and research questions

This paper explores Zimbabwean health practitioners’ understandings of the interconnections between human, animal and environmental health practices and how these understandings can strengthen AMR education frameworks to address antimicrobial misuse.

- What understandings and interpretations of connections between human, animal and environmental health practices emerge in Zimbabwean health practitioners´ discussions of AMR?
- How can these understandings strengthen AMR educational frameworks?

## Materials and methods

This study adopted a qualitative, participatory research design using participatory research workshops.^48–50^ Participatory research workshops focus on knowledge co-creation, drawing on participants´ expertise and experiences. Workshops were guided by principles of participatory research outlined by Bergold & Thomas, Ørngreen & Levinsen and Spinuzzi, creating spaces for engaging with sustainability challenges to promote interdisciplinary knowledge co-creation.^48–52^ Two three-hour workshops were conducted with Zimbabwean health practitioners. Workshops were held during the latter half of 2023 and facilitated by the authors at Parirenyatwa University Hospital and Harare (Sally Mugabe) Hospital. These hospitals were selected because they are principal teaching hospitals in Harare that have a key role in Zimbabwe´s healthcare system and national AMR efforts, as their role as referral hospitals provides care for a broad range of patients within Harare and nationally, while also being where the next generation of the nation’s health practitioners are trained. The participatory workshop method was chosen to enable reflective discussions on AMR and to critically engage with societal factors shaping antimicrobial use^48,49^

Workshops operationalized a case vignette-based workshop tool, where short narratives of real-world situations of antimicrobial use and AMR were shared with participants as stimulations for discussion.^53–55^ Case vignettes were constructed from a combination of previous research on antimicrobial use and AMR in Zimbabwe and contextual insights from practices around antimicrobial use and resistance emergence.^37,43,47,56–61^ Initial drafts were piloted with interdisciplinary research colleagues for linguistic accessibility, clarity and contextual relevance and further refined and developed at research seminars and conferences in Southern Africa.^62–65^ Case vignettes are detailed further in Appendix S1 (available as Supplementary data at *JAC-AMR* Online), together with the semi-structured guiding questions used during the workshops. Questions were designed to facilitate participants’ reflective discussions in small groups and plenary on contextual drivers of antimicrobial use, misuse and AMR (e.g. economic pressures, livestock practices and antimicrobial access). Two vignettes were used, the first focused on the preventive use of broad-spectrum antibiotics (penicillin and erythromycin) in healthy cattle to counter disease risk, reflecting widespread prophylactic practices common in Southern African livestock systems; the second centred on the use of antiretrovirals (ARVs) in small-scale poultry farming to boost production, highlighting documented practices in Zimbabwe where ARVs are redistributed from human patients to poultry under severe economic pressure. While grounded in previous research and contextual insights, vignettes as analytical prompts outline scenarios that may go beyond health practitioners’ everyday experiences, providing opportunities for participants to reflect on and discuss specific antimicrobial practices and associated resistance risks. Together, the vignettes exemplified the impact of antimicrobial use in livestock practices for One health and AMR, with the aim to elicit perceptions, opinions and attitudes on the part of participants. Participants were asked to discuss each vignette as both disciplinary experts through analytical reflections, as well as in relation to clinical and personal experiences. As such, in reflecting on these scenarios from a One health perspective, participants drew on both professional and personal expertise and experiences of antimicrobial use and AMR in Zimbabwe.

Shaped by the framing of the case vignettes, participants´ workshop discussions engaged with commonly used classes of antimicrobials in Zimbabwe. These included broad-spectrum antibiotics such as penicillin and erythromycin, as well as HIV-antiretrovirals, mirroring availability and usefulness in both humans and animals.

The study population included medical doctors, nurses and pharmacists working at the two selected hospitals. At total of 25 health practitioners were recruited using purposive sampling. The sample size was determined pragmatically to allow for in-depth qualitative exploration while making sure the study had diverse representation across professions.

Inclusion criteria required participants to be currently practising health practitioners with routine professional engagement in antimicrobial use and/or AMR-related education, defined through clinical responsibilities such as prescribing, dispensing, administering or counselling patients on antimicrobial use, managing infectious diseases or participating in AMR-related training or stewardship activities.

Diversity was also sought regarding gender, age, and disciplinary background with health practitioners from paediatrics, medicine, surgery and pharmacy, with pharmacists included to strengthen insights regarding drug prescription and dispensing practices impacting access and excessive use of antimicrobials. Participant exclusion criteria included non-practising health practitioners, practitioners who stated before participating that they could not participate in a majority of the workshops, practitioners who did not want to participate in the study, and practitioners who were ill or had at least 5 years without practising medicine. Recruitment of participants at the respective hospital started on 30 June 2023 and ended on 27 July 2023.

The sample size (*n* = 25) was pragmatic to allow for two interactive workshops (*n* = 13 and *n* = 12), enabling small-group breakouts of 3–5 participants for in-depth discussion and exchange between health practitioners. From Parirenyatwa hospital, 10 medical doctors (4 women, 6 men), 2 nurses (2 women) and 1 pharmacist (1 woman) participated. From Harare (Sally Mugabe) hospital, 8 medical doctors (3 women, 5 men), 2 nurses (2 women) and 2 pharmacists (2 women).

**Figure 1.**
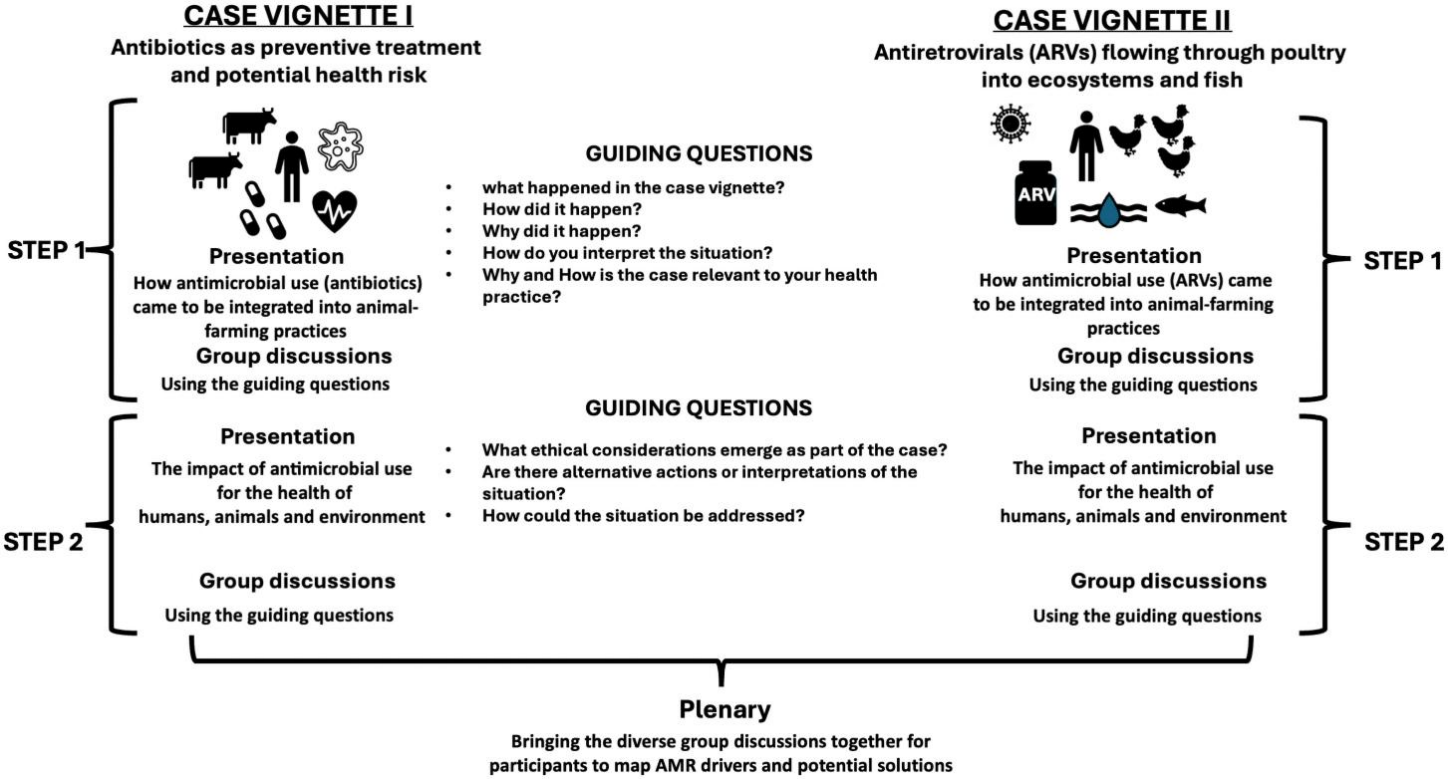
Case vignettes.

Data were generated through facilitated, semi-structured workshop discussions that included both discussions in groups and in plenary. Discussions were audio recorded, with subsequent transcription supported by contemporary field notes. The workshops followed a structured approach: (i) participants were first introduced to AMR as a health challenge and to the purpose of the vignettes; (ii) in small groups, participants analysed vignettes using guiding questions; (iii) groups revisited the vignettes with ethical and policy-oriented questions, including responsibility, trade-offs and systemic opportunities and (iv) a concluding plenary synthesis was held where participants collectively mapped AMR drivers and potential solutions. The workshop design is summarized in Figure 1 and provided further detail in Appendix S1.

This workshop structure encouraged systems thinking and interdisciplinary discussions among the participants, enabling them to draw on their expertise and experiences in reflecting on the case vignettes.

Inductive content analysis method was used together with an analytical framework, detailed below, to guide the analysis process.^66^ Transcripts were reviewed to create an overview of the data, then re-read for further depth. Key sentences were identified as coded as meaningful words and phrases. These were subsequently grouped into categories that, through further re-reading, were developed into analytical themes. Coding was conducted collaboratively by the authors through comparison and joint discussion, supported by field notes, to ensure consistency and to deepen interpretation and analysis. Participants’ voices were important in the analysis, and representative quotations are used throughout the results section to support the findings. As part of the analysis, we considered how the vignettes impacted the direction of discussions by being attentive to quotations resulting from discussions on the vignette narratives in contrast with those resulting from participants’ reflection on professional experiences. The focus of the analysis was on health practitioners’ understandings and sense-making around antimicrobial movements across domains rather than empirical evidence of redistribution practices.

Ethical approval was obtained from the Medical Research Council of Zimbabwe (MRCZ/A/2920) and from the institutions’ ethical review boards (JREC Ref: 212/2022). Informed consent was secured from all participants, and confidentiality was maintained throughout. To maintain confidentiality, participants were coded with numbers, and no participant names were recorded as part of data collection. Throughout the study, the global code of conduct for research in resource-poor settings was adhered to.^67^

## Results

The results from the AMR workshop discussions reveal a complex and interconnected set of factors that participants perceived as being linked with the rising prevalence of drug-resistant infections in Zimbabwe. The dominant theme resulting from the participants’ discussions was antimicrobial redistribution, framed by the participants as the moving of antimicrobials from their intended clinical use and across human, animal and environmental domains. Participants outlined as part of this broader theme three interconnected but analytically separate processes of redistribution: (i) redistribution to animals, understood as the perceived moving of antimicrobials from human patients to animals, (ii) redistribution to humans, described as the informal sharing of antimicrobials between humans and (iii) redistribution to environments, understood as the unintentional contamination of environments through waste, manure and runoff.

### Redistribution to animals

The sub-theme of redistributing antimicrobials, focused on the participants’ discussions regarding antimicrobial use in livestock, was prompted by the case vignette narratives, which participants connected to broader AMR challenges. Practitioners expressed concern over what they understood as unregulated antimicrobial use in livestock, including reflections on the risks, outlined in the vignettes, of ARVs being fed to poultry to accelerate growth, as illustrated in the quote below:

‘The poultry farmer was using the ARVs to make the birds grow quicker and bigger’. (Participant 4, reflecting on the vignettes, Parirenyatwa hospital).

Furthermore, participants highlighted how antimicrobial use was understood as shifting from a question of health to a question of business and economy, as seen in the following quote,

The challenge is that farmers don’t see antibiotics as a health issue; they see them as a business tool. They are looking for faster-growing chickens, bigger cows, more milk. (Participant 2, reflecting on own experience, Parirenyatwa hospital)

Other participants emphasized how the vignette scenarios engaged with perceived risks of patent compliance with antimicrobial prescriptions:

The redistribution of antiretroviral drugs to chickens by small-scale farmers, which can lead to non-compliance with antiretroviral drug regimes and the need for more antibiotics (Participant 4, reflecting on the vignettes, Harare hospital)

Participants highlighted in their discussions concerns about the possible health impacts of eating animals that have been fed antimicrobials, including the risks of resistance, as illustrated by the reflections in relation to both the vignettes and their own experiences:

If we eat chickens that have been given ARVs, what happens to us in the long run? We need research on the long-term impact. (Participant 1, reflecting on the vignettes, Parirenyatwa hospital)

Participants expressed concerns regarding the perceived links between antimicrobial use in farming animals and its long-term impacts on human health. Participants also connected the antimicrobial misuse outlined in the vignettes to intentional behaviour they reported having experienced in professional and community settings:

People are corrupt. People, because of much that they're getting, they can do anything. As long as they feel that there is a gap, that they can misuse. (Participant 5, reflecting on own experience, Harare hospital).

In participants’ discussions redistribution of antimicrobials to livestock was understood as posing a potential risk, which they viewed as possibly contributing to the spread of resistance.

### Redistribution to humans

Participants outlined their understanding of redistribution as potentially occurring between humans, with medication sharing described as being common among family, friends and community members. These discussions were largely grounded in participants’ professional experience with medication sharing outlined as common, particularly among individuals facing financial hardship and unable to afford medical consultations:

You take your prescription, let’s say for a cough, then your neighbour starts to have a similar cough. But because you know that your neighbour is a bit financially challenged, you then dispense your own prescription to your neighbour as a way of trying to help, not knowing the implications of your actions. (Participant 4, reflection on own experience, Parirenyatwa hospital)

Participants highlighted how redistribution practices have diverse reasons, including a lack of awareness as well as solidarity with family and community:

Ignorance is there, we cannot ignore it, it's there. For some, they did not even know. If you all knew that if I give him my tablets that I have taken, maybe they will have a negative impact. […] They will be doing it in utmost good faith, they think, no, I'm actually assisting my brother, I'm actually assisting my sister or my relative, or my neighbour, not knowing that they are actually creating something else. (Participant 2, reflecting on own experience, Harare hospital)

There is also a strong cultural perception of medications as universally beneficial, as in the following quote,

At funerals or gatherings, the elderly will say, ‘Can I also have that medication you are taking?’ without even knowing what it is for. (Participant 2, reflecting on own experience, Parirenyatwa hospital)

### Redistribution environments

Engagement with the vignettes also enabled participants to reflect on the impacts of antimicrobials on the environment.

Even the population down the river is now affected because of the antimicrobials that are coming from the farming. Yeah, it has a lot of impact to the humans and the ecosystem. Because it's often like we might not think that if we give the chickens some ARVs that the fish in the river downstream is going to be affected, that it is to some degree a free product (Participant 4, reflecting on the vignettes, Harare hospital).

Participants also linked the vignette discussions to professional experiences and insights regarding perceived environmental contamination pathways:

When you take the excrement and put it in the fields now you are affecting the microbes and if maybe there is rain and the water flows into the rivers and into the wells. Then you are affecting the human consumption (Participant 1, reflecting on own experience, Harare hospital).

Participants interpreted antimicrobial use as linked to a number of avenues for environmental contamination, including wastewater and agricultural runoff, and expressed concern regarding what they perceived as potential implications for human health.

### Drivers of antimicrobial redistribution

Participants identified key drivers that they linked to antimicrobial redistribution, including financial pressures, social solidarity, lack of awareness and limited veterinary oversight. These drivers were discussed by the participants as potentially exacerbating indiscriminate antimicrobial use:

We need to talk as a group of different organisations to regulate the use of these medications in animals before the situation worsens. (Participant 5, reflecting on own experience, Parirenyatwa hospital).

In addition, concerns were raised by participants regarding non-compliance when discussing the vignette scenarios:

The challenges of non-compliance with antiretroviral drug regimes and the subsequent need for more antibiotics are highlighted, especially in a pressured economy like Zimbabwe (Participant 3, reflecting on vignette, Harare hospital).

Workshop participants highlighted how they understood the drivers of antimicrobial redistribution as creating feedback loops, which they viewed as reinforcing the challenge of AMR:

If people are taking this medication and the kids are taking this medication, because we have kids already, the AMR is starting, that’s the start of it. And the second thing is we have found animals coming back to the people, and that resistance will continue. (Participant 5, reflecting on own experience, Parirenyatwa hospital).

Throughout the workshop discussions, participants articulated their understanding of links between antimicrobial use across human, animal and environmental domains, and how they perceived these interconnections impacting the development of resistance.

## Discussion

This paper explored how Zimbabwean health practitioners’ understandings and sense-making regarding how antimicrobials were perceived as moving across move across human, animal and environmental domains and their impact on AMR. Workshop discussions were based on two case vignettes of antimicrobial use in small-scale farming livestock. The central finding was that participants emphasized the redistribution of antimicrobials across humans, animals and environments, which they described as potentially creating a reinforcing cycle of resistance. This redistribution encompassed three analytically separate but interconnected processes as discussed by the participants: (i) patients using, providing or selling antimicrobials for use in livestock, (ii) informal sharing of medicines among family and community members and (iii) contamination of environments through livestock waste and agricultural runoff. Participants discussed how antimicrobial redistribution may contribute to drug-resistant infections, particularly through the way people share medicines with relatives or animals, highlighting how medicines could be diverted to family, community and for animal use. These interpretations reflect previous research outlining how the spread of antimicrobials can reinforce resistance cycles between humans, animals and environments.^28,29^ Redistribution was thus used as an analytical framing concept to highlight how participants understood antimicrobial movements across human, animal and environmental domains beyond intended clinical use, with the perceived risk of driving resistance.

**Figure 2.**
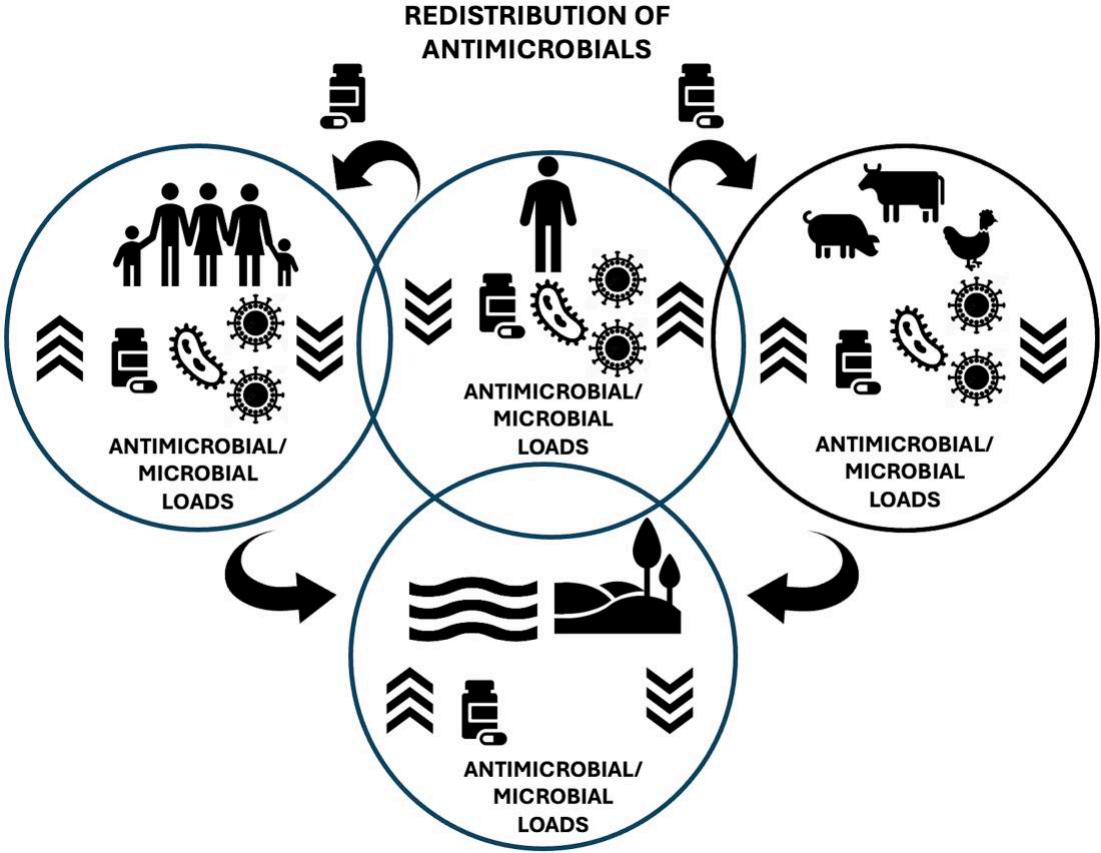
Redistribution of antimicrobials.

Participants also described, especially based on the vignettes, how the use of antimicrobials in farming was understood as a strategy to make chickens grow faster, linking antimicrobial misuse to socio-economic pressures and the need to promote livestock growth. Meanwhile, participants expressed concerns that consuming chickens raised on ARVs may result in exposure to residual drugs or resistant pathogens, reflecting perceived risk, which they viewed as increasing the risk of developing drug-resistant infections. Environmental contamination from household and livestock waste was framed by participants as difficult to avoid because of what they perceived as limited proper disposal options, linking inadequate waste management to contamination and perceived risks of spreading AMR back to humans. This illustrates how participants viewed resistant microbes as moving between humans, animals and the environment, creating what they described as a self-reinforcing cycle. The identified themes highlight, in the participants’ view, the importance for AMR education to engage with the complexities of drug use, compliance and the interconnectedness of human, animal and environmental health. These findings illustrate how health practitioners link everyday practices to healthcare systems, livestock farming and environments, framing antimicrobial redistribution as a perceived contributing driver of AMR, as outlined in Figure 2.

The overlapping circles represent interconnected human (individual and community), animal, and environmental domains with antimicrobials not limited to the domain in which they are intended to be used, but that move across domains. Curved arrows are used to illustrate redistribution processes, such as wastewater, agricultural runoff, food systems and environmental contamination. Participants outlined these processes as generating feedback loops where redistributed antimicrobials were understood by participants to contribute to resistance, which can present a risk for human health.

The environmental domain at the bottom is presented as a space of antimicrobial accumulation as well as a source of potential exposure for humans and animals. Within the circles, arrows represent the balance between antimicrobials and microbial loads within the specific domain. Pill symbols illustrate antimicrobial inputs; microbial icons represent affected microbial communities. The figure outlines antimicrobial redistribution as a central process through which participants understand systemic and reinforcing antimicrobial resistance dynamics.

Participants described antimicrobial redistribution as often motivated by social and economic factors such as solidarity with family and community members and economic survival strategies. This emphasis on everyday practices of sharing and reselling antimicrobials aligns with previous research.^45,46^ Redistribution practices were often framed by the participants as well-intentioned but problematic, as they were viewed as increasing risks of resistance and health complications for the wider community. These practices were perceived as posing serious risks to human health, especially when framed as concerns for drug-resistant bacteria entering the food chain. For example, participants expressed concern that these practices could potentially contribute to the HIV virus developing resistance against first-generation ARVs. Redistribution to animals was linked to food security and household income, with farmers described as seeking to maximize livestock growth, which is in line with research on the role of agriculture in increasing AMR spread.^68^ Redistribution to family and community members was linked to difficulties accessing healthcare due to cost and availability as has been highlighted in previous research.^69,70^ Furthermore, redistribution to the environment was understood in terms of an unintentional but often inevitable outcome of limited waste management and water treatment infrastructure, reflecting findings regarding environmental reservoirs of resistance.^71^ Studies in neighbouring countries have highlighted how exposure to low concentrations of antibiotics in water sources can accelerate antimicrobial resistance.^72^ The contamination of Zimbabwe’s water systems, agricultural land and aquatic life (e.g. Tilapia fish showing liver damage) raises concerns about AMR transmission from the environment back to humans.^73^

While Zimbabwe’s AMR policy set clear guidelines and goals for antimicrobial stewardship, antimicrobial redistribution highlights how the limited regulatory enforcement and monitoring, inconsistent veterinary and agricultural extension support, as perceived by the participants, contribute to challenges in controlling the use of clinically intended antimicrobials in animals.^18,20–22^ Gaps in AMR policy implementation were understood by the participants as reinforcing the redistribution processes detailed in this paper, as antimicrobials are allowed to circulate through the medical and veterinary health system.

These findings align with previous One health research that highlights how resistance is not limited to healthcare settings but circulates with impacts for humans, animals and environments.^3,12,23,24,74,75^ However, this study contributes a health practitioner perspective, by examining how health practitioners understand and make sense of redistribution practices. A crucial insight was how health practitioners recognized antimicrobials as both medicines and economic resources. This has significant implications for the functions of antimicrobials in society, shaping how they are valued and used in everyday life. These multiple roles of antimicrobials illustrate a key consideration for AMR education efforts.

A key implication, based on participants’ discussions, is that AMR education in Zimbabwe should extend beyond awareness-raising and correct prescribing in clinical settings to address antimicrobial redistribution and its social and economic drivers. While communication regarding responsible antimicrobial use remains essential, AMR education should acknowledge how pressures of economic survival, social solidarity, and food security shape antimicrobial practices. As such, redistribution cannot be addressed through awareness campaigns alone. Educational efforts should thus engage with health practitioners (medical doctors, nurses and pharmacists), who prescribe and counsel patients around antimicrobials. This could include case-based learning of how to discuss and address antimicrobial redistribution, medicine sharing, use in livestock and proper disposal, in interactions with patients. Small-scale farmers were identified by participants as an important group to engage, as farmers were perceived to potentially redistribute antimicrobials intended for clinical use to farming animals if put under social and economic pressure. Education efforts could be integrated with veterinary and agricultural extension services. To effectively integrate antimicrobial redistribution would necessitate the inclusion of social, economic and environmental dimensions as part of existing AMR stewardship efforts. Drawing on this paper, this could be achieved using case vignettes to enable engagement with value conflicts, food security challenges, and environmental resistance pathways. Meanwhile, integration is dependent on available resources, clinical, veterinary and environmental monitoring, as well as the state of waste infrastructure. This highlights how a coordinated One health approach is key for AMR education to align and collaborate with broader policy efforts, thus going beyond individual behavioural change.

A second implication is the necessity for transdisciplinary collaborations across medical health, veterinary and environmental sectors. Participants’ discussions highlighted gaps in monitoring across these domains, with how limited veterinary support was understood by participants as potentially enabling the redistribution of antimicrobials to livestock, including for prophylactic and growth-promoting purposes. In parallel, participants highlighted perceived weaknesses in pharmaceutical waste regulation and monitoring as a key contributing factor to antimicrobial redistribution into environments. Drawing on the One health approach, AMR education could include veterinarians and small-scale farmers to identify pathways to bridge this gap of redistribution to animals, while the involvement of policy makers and regulators would be crucial to address the flow of antimicrobials into environments.

Situating redistribution within a One health framework shows how structural factors, including poverty, socio-economic marginalization, uneven healthcare access and limited regulation, reinforce AMR challenges in Zimbabwe. Effective and contextually relevant AMR education would thus require active engagements with socio-economic and structural conditions, multisectoral collaborations and strengthening policy implementation.

The paper includes a number of research limitations, among which the sample of participating health practitioners being limited to two urban hospitals. As noted, these were chosen for their key roles as both referral hospitals, receiving patients both locally in Harare and nationally, and as teaching hospitals, educating the next generation of health practitioners. Meanwhile, they may not cover the perspectives and experiences of the more rural contexts where many of the redistribution practices of antimicrobials from patients to livestock and to other family and community members occur. In addition, while the case vignettes were important in supporting the structure of the discussions, they also directed and could have, to a certain degree, impacted the themes that emerged in the participant discussions. The identified themes should thus be viewed as illustrating participants’ sense-making and reflective discussions in response to the vignette prompts, rather than as evidence of how common processes of antimicrobial redistribution are. While considering these limitations, the vignette-based workshop approach aligned with the aim to explore health practitioners’ understandings of the interconnections between human, animal and environmental health practices. Using case scenarios as an analytical tool can be useful in prompting and encouraging participants’ assumptions and values, as well as identifying tensions related to antimicrobial practices which might not surface if discussions are limited to everyday practices.

Future research could include small-scale farmers, rural health practitioners, veterinarians and environmental experts to deepen understandings of the antimicrobial redistribution dynamics identified in this paper.

The paper illustrates how antimicrobial redistribution is understood by health practitioners as contributing to AMR in Zimbabwe. By identifying and exploring, together with health practitioners, how antimicrobials move through everyday practices across humans, animals and the environment, the paper emphasizes the need to engage with redistribution practices in AMR education. Setting AMR education within a One health framework would strengthen its ability to address antimicrobial misuse and limit the development and spread of resistance.

## Supplementary Material

dlag019_Supplementary_Data

## Data Availability

The data supporting the results of this study are available from the corresponding author upon reasonable request.

## Supplementary data

Appendix S1 is available as Supplementary data at *JAC-AMR* Online.

## References

[1] Taneja N, Sethi S, Tahlan AK et al Introductory Chapter: Stepping into the Post-Antibiotic Era—Challenges and Solutions. Antimicrobial Resistance-A Global Threat. IntechOpen, 2019.

[2] Davies J, Davies D. Origins and evolution of antibiotic resistance. Microbiol Mol Biol Rev 2010; 74: 417–33. 10.1128/MMBR.00016-10 20805405 PMC2937522

[3] Hernando-Amado S, Coque TM, Baquero F et al Defining and combating antibiotic resistance from one health and global health perspectives. Nat Microbiol 2019; 4: 1432–42. 10.1038/s41564-019-0503-9 31439928

[4] Ahmed SK, Hussein S, Qurbani K et al Antimicrobial resistance: impacts, challenges, and future prospects. J Med Surg Public Health 2024; 2: 100081. 10.1016/j.glmedi.2024.100081

[5] Keenan K, Silva Corrêa J, Sringernyuang L et al The social burden of antimicrobial resistance: what is it, how can we measure it, and why does it matter? JAC Antimicrob Resist 2025; 7: dlae208. 10.1093/jacamr/dlae208 40065828 PMC11891513

[6] Naghavi M, Vollset SE, Ikuta KS et al Global burden of bacterial antimicrobial resistance 1990–2021: a systematic analysis with forecasts to 2050. Lancet 2024; 404: 1199–226. 10.1016/S0140-6736(24)01867-1 39299261 PMC11718157

[7] Cole J, Eskdale A, Paul JD. Tackling AMR: a call for a (n even) more integrated and transdisciplinary approach between planetary health and earth scientists. Challenges 2022; 13: 66. 10.3390/challe13020066

[8] Sartorius B, Gray AP, Weaver ND et al The burden of bacterial antimicrobial resistance in the WHO African region in 2019: a cross-country systematic analysis. Lancet Glob Health 2024; 12: e201–16. 10.1016/S2214-109X(23)00539-9 38134946 PMC10805005

[9] Littmann J, Viens AM, Silva DS. The super-wicked problem of antimicrobial resistance. In: Ethics and Drug Resistance: Collective Responsibility for Global Public Health, Springer, 2020; 5: 421–43.

[10] Matthiessen LE, Hald T, Vigre H. System mapping of antimicrobial resistance to combat a rising global health crisis. Front Public Health 2022; 10: 816943. 10.3389/fpubh.2022.816943 35784220 PMC9249020

[11] Rickard H, Watkin S, Baldwin N et al Antimicrobial resistance as a super wicked problem: how do we engage the public to be part of the solution. Infect Prev Pract 2023; 5: 100314. 10.1016/j.infpip.2023.100314 38107239 PMC10724478

[12] Abia ALK, Essack SY. Antimicrobial Research and One Health in Africa. Springer, 2023.

[13] Charani E, Mendelson M, Ashiru-Oredope D et al Navigating sociocultural disparities in relation to infection and antibiotic resistance-the need for an intersectional approach. JAC Antimicrob Resist 2021; 3: dlab123. 10.1093/jacamr/dlab123 34604747 PMC8485076

[14] Manyau S . An Ethnography of Antibiotics and Antimicrobial Resistance, in the Lives of Medicine Providers, Residents, and sex Workers. London School of Hygiene & Tropical Medicine, 2024.

[15] Chitungo I, Dzinamarira T, Nyazika TK et al Inappropriate antibiotic use in Zimbabwe in the COVID-19 era: a perfect recipe for antimicrobial resistance. Antibiotics (Basel) 2022; 11: 244. 10.3390/antibiotics11020244 35203846 PMC8868384

[16] Olaru ID, Ferrand RA, Yeung S et al Knowledge, attitudes and practices relating to antibiotic use and resistance among prescribers from public primary healthcare facilities in Harare, Zimbabwe. Wellcome Open Res 2021; 6: 72. 10.12688/wellcomeopenres.16657.1 37780956 PMC10534082

[17] Dixon J, MacPherson EE, Nayiga S et al Antibiotic stories: a mixed-methods, multi-country analysis of household antibiotic use in Malawi, Uganda and Zimbabwe. BMJ Glob Health 2021; 6: e006920. 10.1136/bmjgh-2021-006920 PMC8628329 34836911

[18] MOHCC . Zimbabwe One Health Antimicrobial Resistance National Action Plan 2017-2021. Government of Zimbabwe, 2017.

[19] WHO . Global Action Plan on Antimicrobial Resistance. WHO, 2015.

[20] Mickelsson M, Oljans E. Agile policies for antimicrobial resistance: a contextual approach to sustainable health challenges. Glob Public Health 2025; 20: 2522913. 10.1080/17441692.2025.2522913 40552562

[21] Mishra KM, Muraya AT, Williams N et al Bridging AMR knowledge gaps and improving policy implementation: a perspective on the role of community engagement in Africa. Front Public Health 2025; 13: 1668830. 10.3389/fpubh.2025.1668830 41358212 PMC12678311

[22] Matope G, Mugabe P, Kapungu F et al One health landscape in Zimbabwe: current status, challenges and opportunities for institutionalisation. One Health Cases 2024; 2024: ohcs20240017. 10.1079/onehealthcases.2024.001.

[23] Zinsstag J, Meyer JM, Bonfoh B et al One health in human-environment systems: linking health and the sustainable use of natural resources. CABI One Health 2024; 3.

[24] Meisner J, McLeland-Wieser H, Traylor EE et al Relational one health: a more-than-biomedical framework for more-than-human health, and lessons learned from Brazil, Ethiopia, and Israel. One Health 2024;18: 100676. 10.1016/j.onehlt.2024.100676 39010955 PMC11247262

[25] Bengtsson-Palme J, Abramova A, Berendonk TU et al Towards monitoring of antimicrobial resistance in the environment: for what reasons, how to implement it, and what are the data needs? Environ Int 2023; 178: 108089. 10.1016/j.envint.2023.108089 37441817

[26] Aenishaenslin C, Häsler B, Ravel A et al Evaluating the integration of one health in surveillance systems for antimicrobial use and resistance: a conceptual framework. Front Vet Sci 2021; 8: 611931. 10.3389/fvets.2021.611931 33842569 PMC8024545

[27] Pruden A, Larsson DJ, Amézquita A et al Management options for reducing the release of antibiotics and antibiotic resistance genes to the environment. Environ Health Perspect 2013; 121: 878–85. 10.1289/ehp.1206446 23735422 PMC3734499

[28] Robinson TP, Wertheim HF, Kakkar M et al Animal production and antimicrobial resistance in the clinic. The Lancet 2016; 387: e1–3. 10.1016/S0140-6736(15)00730-8 26603925

[29] Van Boeckel TP, Glennon EE, Chen D et al Reducing antimicrobial use in food animals. Science 2017; 357: 1350–2. 10.1126/science.aao1495 28963240 PMC6510296

[30] WHO . Global Antimicrobial Resistance and use Surveillance System (GLASS) Report 2022. World Health Organization, 2022.

[31] Shawoo Z, Maltais A, Dzebo A et al Political drivers of policy coherence for sustainable development: an analytical framework. Environ Policy Gov 2023; 33: 339–50. 10.1002/eet.2039

[32] Chatora R, Tumusiime P. Health Sector Reform and District Health Systems. WHO, 2004.

[33] Pokharel S, Raut S, Adhikari B. Tackling antimicrobial resistance in low-income and middle-income countries. BMJ Special J 2019; 4: e002104. 10.1136/bmjgh-2019-002104 PMC6861125 31799007

[34] Makuwerere Dube L . Command agriculture and food security: an interrogation of state intervention in the post-fast track land redistribution era in Zimbabwe. J Asian Afr Stud 2021; 56: 1626–43. 10.1177/0021909620979330

[35] Sulis G, Sayood S, Gandra S. Antimicrobial resistance in low-and middle-income countries: current status and future directions. Expert Rev Anti Infect Ther 2022; 20: 147–60. 10.1080/14787210.2021.1951705 34225545

[36] Richards S, Knight-Jones T, Angombe S et al Towards institutionalization of one health in eastern and Southern Africa. One Health Cases 2024; 2024: ohcs20240007. 10.1079/onehealthcases.2024.0007

[37] Gufe C, Jambwa P, Bare W et al Antimicrobial use and antimicrobial resistance in the poultry value chain in Zimbabwe: a review. Tanzan Vet J 2023; 38.

[38] Lukwa AT, Siya A, Zablon KN et al Socioeconomic inequalities in food insecurity and malnutrition among under-five children: within and between-group inequalities in Zimbabwe. BMC Public Health 2020; 20: 1–11. 10.1186/s12889-020-09295-z 32753035 PMC7406388

[39] Palanco Lopez P, Chandler CI. Histories of antibiotics: a one health account of the arrival of antimicrobial drugs to Zimbabwe, Malawi and Uganda. Report for the improving human health flagship initiative, agriculture for nutrition and health research programme, CGIAR. London School of Hygiene & Tropical Medicine, 2020 10.17037/PUBS.04658867.

[40] Sono TM, Yeika E, Cook A et al Current rates of purchasing of antibiotics without a prescription across sub-Saharan Africa; rationale and potential programmes to reduce inappropriate dispensing and resistance. Expert Rev Anti Infect Ther 2023; 21: 1025–55. 10.1080/14787210.2023.2259106 37740561

[41] Hedman HD, Vasco KA, Zhang L. A review of antimicrobial resistance in poultry farming within low-resource settings. Animals 2020; 10: 1264. 10.3390/ani10081264 32722312 PMC7460429

[42] Candel JJ . The expediency of policy integration. Policy Studies 2021; 42: 346–61. 10.1080/01442872.2019.1634191

[43] Munengwa A, Nation C, Alban M. Perceptions and practices on antimicrobial use by the farmers of the chikomba district, Zimbabwe. Aceh J Anim Sci 2020; 5: 73–80. 10.13170/ajas.5.2.16713

[44] Mwansa M, Mukuma M, Mulilo E et al Determination of antimicrobial resistance patterns of Escherichia coli isolates from farm workers in broiler poultry production and assessment of antibiotic resistance awareness levels among poultry farmers in Lusaka, Zambia. Front Public Health 2023; 10: 998860. 10.3389/fpubh.2022.998860 36703831 PMC9871586

[45] Ikhimiukor OO, Odih EE, Donado-Godoy P et al A bottom-up view of antimicrobial resistance transmission in developing countries. Nat Microbiol 2022; 7: 757–65. 10.1038/s41564-022-01124-w 35637328

[46] Okeke IN, Laxminarayan R, Bhutta ZA et al Antimicrobial resistance in developing countries. Part I: recent trends and current status. Lancet Infect Dis 2005; 5: 481–93. 10.1016/S1473-3099(05)70189-4 16048717

[47] Dixon J, Manyau S, Kandiye F et al Antibiotics, rational drug use and the architecture of global health in Zimbabwe. Soc Sci Med 2021; 272: 113594. 10.1016/j.socscimed.2020.113594 33529937

[48] Bergold J, Thomas S. Participatory research methods: a methodological approach in motion. Hist Soz Forsch 2012; 37: 191–222. 10.12759/hsr.37.2012.4.191-222

[49] Spinuzzi C . The methodology of participatory design. Tech Commun 2005; 52: 163–74. http://www.jstor.org/stable/43089196

[50] Ørngreen R, Levinsen KT. Workshops as a research methodology. Electronic Journal of E-learning 2017; 15: 70–81. https://vbn.aau.dk/ws/portalfiles/portal/257686207/_rngreen_Levinsen_Workshop_as_a_Research_methodology_ejel_volume15_issue1_article569.pdf

[51] Head BW . Wicked Problems in Public Policy: Understanding and Responding to complex Challenges. Springer Nature, 2022.

[52] Head BW . Political Governance of Wicked Problems. Wicked Problems in Public Policy: Understanding and Responding to Complex Challenges. Springer, 2022; 37–60.

[53] Carlile PR . A pragmatic view of knowledge and boundaries: boundary objects in new product development. Organization science 2002; 13: 442–55. 10.1287/orsc.13.4.442.2953

[54] Sheringham J, Kuhn I, Burt J. The use of experimental vignette studies to identify drivers of variations in the delivery of health care: a scoping review. BMC Med Res Methodol 2021; 21: 81. 10.1186/s12874-021-01247-4 33888077 PMC8061048

[55] Mickelsson M, Oljans E. Case vignettes-based workshops as a tool to facilitate interdisciplinary engagement with complex problems in sustainability education. Discov Sustain 2025; 6: 687. 10.1007/s43621-025-01631-w

[56] Caudell MA, Dorado-Garcia A, Eckford S et al Towards a bottom-up understanding of antimicrobial use and resistance on the farm: a knowledge, attitudes, and practices survey across livestock systems in five African countries. PLoS One 2020; 15: e0220274. 10.1371/journal.pone.0220274 31978098 PMC6980545

[57] CDDEP . Situational analysis of antimicrobial use and resistance in humans and animals in Zimbabwe. CDDEP, 2017 https://cddep.org/wp-content/uploads/2017/10/SITUATION-ANALYSIS-OF-ANTIMICROBIAL-USE-AND-RESISTANCE-IN-HUMANS-AND-ANIMALS-IN-ZIMBABWE-1.pdf.

[58] Moyo B, Ndlovu S, Moyo S et al Alternative remedies and approaches used by resources-challenged farmers in the management of cattle black-leg disease in umzingwane district, matabeleland south, Zimbabwe. Int J Livest Prod 2014; 6(6): 97–102. 10.5897/IJLP2013.0198

[59] Moyo BH, Thow AMT. Fulfilling the right to food for South Africa: justice, security, sovereignty and the politics of malnutrition. World Nutrition 2020; 11: 112–52. 10.26596/wn.2020113112-152

[60] Swiswa S, Obonyo M, Dube K et al Measurement of antibiotic use on poultry farms in Zimbabwe: Evaluation of a tool and procedures. Fleming Fund Fellowship (LSHTM) Reports, 2022 10.17037/PUBS.04668058.

[61] Swiswa S, Pinto CJ, Chandler CI. Antimicrobial Use/Consumption Surveillance in Zimbabwe. Desk Review Report. London School of Hygiene & Tropical Medicine, 2022 10.17037/PUBS.04668339.

[62] Converse L, Barrett K, Rich E et al Methods of observing variations in physicians’ decisions: the opportunities of clinical vignettes. J Gen Intern Med 2015; 30: 586–94. 10.1007/s11606-015-3365-8 PMC4512963 26105672

[63] Kathiresan J, Patro BK. Case vignette: a promising complement to clinical case presentations in teaching. Educ Health 2013; 26: 21–4. 10.4103/1357-6283.112796 23823669

[64] Payton KS, Gould JB. Vignette research methodology: an essential tool for quality improvement collaboratives. Healthcare 2022; 11: 7. 10.3390/healthcare11010007 36611468 PMC9818599

[65] Veloski J, Tai S, Evans AS et al Clinical vignette-based surveys: a tool for assessing physician practice variation. Am J Med Qual 2005; 20: 151–7. 10.1177/1062860605274520 15951521

[66] Graneheim UH, Lundman B. Qualitative content analysis in nursing research: concepts, procedures and measures to achieve trustworthiness. Nurse Educ Today 2004; 24: 105–12. 10.1016/j.nedt.2003.10.001 14769454

[67] Trust . Global Code of Conduct for Research in Resource-Poor Settings. Trust, 2018.

[68] Goutard FL, Bordier M, Calba C et al Antimicrobial policy interventions in food animal production in South East Asia. BMJ 2017; 358: j3544. 10.1136/bmj.j3544 28874351 PMC5598294

[69] Gelband H, Laxminarayan R. Tackling antimicrobial resistance at global and local scales. Trends Microbiol 2015; 23: 524–6. 10.1016/j.tim.2015.06.005 26338444

[70] Llor C, Bjerrum L. Antimicrobial resistance: risk associated with antibiotic overuse and initiatives to reduce the problem. Ther Adv Drug Saf 2014; 5: 229–41. 10.1177/2042098614554919 25436105 PMC4232501

[71] Kusi J, Ojewole CO, Ojewole AE et al Antimicrobial resistance development pathways in surface waters and public health implications. Antibiotics 2022; 11: 821. 10.3390/antibiotics11060821 35740227 PMC9219700

[72] Gwenzi W, Ngaza N, Marumure J et al Occurrence and Health Risks of Antibiotic Resistance in African Aquatic Systems. Springer International Publishing AG, 2023; 107–59.

[73] Moffo F, Ndebé MMF, Tangu MN et al Antimicrobial use, residues and resistance in fish production in Africa: systematic review and meta-analysis. BMC Vet Res 2024; 20: 307. 10.1186/s12917-024-04158-w 38987775 PMC11234786

[74] Zinsstag J, Kaiser-Grolimund A, Heitz-Tokpa K et al Advancing one human–animal–environment health for global health security: what does the evidence say? The Lancet 2023; 401: 591–604. 10.1016/S0140-6736(22)01595-1 36682371

[75] Destoumieux-Garzón D, Mavingui P, Boetsch G et al The one health concept: 10 years old and a long road ahead. Front Vet Sci 2018; 5: 14. 10.3389/fvets.2018.00014 29484301 PMC5816263

