# Supplementary material for: Antimicrobial redistribution: health practitioner One health perspectives on antimicrobial use in livestock for antimicrobial resistance education in Zimbabwe
Source: JAC Antimicrob Resist. 2026 Feb 17;8(1):dlag019. doi: 10.1093/jacamr/dlag019 (PMC12910502; doi:10.1093/jacamr/dlag019)
Supplement: dlag019_Supplementary_Data [file dlag019_supplementary_data.docx]

## Appendix 1 – Case vignettes and workshop questions

The two case vignettes engage with how antimicrobial practices and misuse illustrate intersections between human, animal, and environmental health. Each vignette highlights pathways through which everyday practices of small-scale farmers influence and are influenced by broader health systems.

The first vignette draws on research and reports highlighting how antibiotics are routinely used in livestock practices such as cattle production across Southern Africa. These practices involve farmers mixing antibiotics into the feed of healthy animals to prevent potential infection, a practice that has been recognized in research and reporting as contributing to AMR risks.

The second vignette illustrates practices documented in research and reporting from the Southern African region of farmers re-distributing antiretroviral drugs (originally intended for treating HIV/AIDS human patients) to livestock, specifically poultry to promote growth. This case vignette underscores how human health interventions can spill over into animal health practices, with consequences for environmental health and driving AMR.

Together, these vignettes highlight One health dimensions of AMR through the redistribution of antimicrobials from human patients to livestock, environments and broader community. AMR and antimicrobial practices are thus situated within the lived realities of farmers, engaging with biomedical and socio-economic drivers of antimicrobial misuse.

**Case vignette 1: Antibiotics as preventive treatment and potential health risk**

Thokozani has recently taken over responsibility for the family beef cattle farm from his father and while eager to develop it and grow the herd he is also concerned about the health of the cattle and the risk of disease as the extended family relies on the beef cattle for important dietary protein as well as a source for profit. Within Zimbabwe, there are many endemic infectious diseases that affect cattle, and that could threaten the food security and health of the community.

During the first year of running the farm, there are outbreaks of Black leg in the region, severe cattle disease caused by the bacteria *Clostridium chauvoei* affecting young and responsible for a significant part of cattle mortalities in Zimbabwe. The majority of antibiotics in Southern Africa are used in cattle and poultry farming often as part of proactive measures. Although vaccines are encouraged by the Department of Veterinary Services (DVS), the use of antibiotics remains the major preventive and control strategy for most bacterial infections in cattle in Zimbabwe. As such, the idea of using antibiotics to prevent potential disease is close for Thokozani. By mixing the antibiotic *penicillin* into the feed of his healthy cattle, he hopes to safeguard the food security and health of his family. It is an initial success, none of his cattle is infected, and the use of antibiotics in the feed increases the growth of the cattle, making the farm more profitable.

***Case vignette 1 workshop discussion, round 1***

- What happened in the case vignette?
- How did it happen?
- Why did it happen?
- How do you interpret the situation?
- Why and How is the case relevant to your health practice?

***Case vignette 1 continued***

Encouraged, Thokozani continues mixing antibiotics, penicillin and over time other antibiotics (erythromycin**)**, in the cattle feed until he hears about an outbreak of a multi-resistant bacterial infection at the local clinic. The bacteria causing the infection turns out to be resistant to antibiotics available at the clinic and patients have to be sent for intensive care in the regional hospital. The Health Department and the DVS launch investigations to track the origin of the multi-resistant mutation of the bacteria in both the community and the animal population. At a meeting with the community, the government reports that resistant bacteria is traced to nearby populations of African buffalo and local cattle populations, including those of Thokozani and neighbouring farmers.

The DVS is especially concerned as they refer to research that shows how the mixing of antibiotics into feed to prevent infections such as Black leg can be a driver for antimicrobial resistance (AMR). Furthermore, the representatives caution the community members from eating unwashed vegetables as *E. coli* bacteria resistant to antibiotics, among other *penicillin*, were found in soil samples taken from vegetable plots fertilised with manure from the local cattle farms.

***Case vignette 1 workshop discussion, round 2***

*Additional questions for discussion*

- What ethical considerations emerge as part of the case?
- Are there alternative actions or interpretations of the situation?
- How could the situation be addressed?

**Case vignette 2: ARVs flowing through poultry into ecosystems and fish**

Chikondi has worked for a long-time raising poultry at his small-scale farm for profit to pay for school fees for his children. Recently the cost-per-kilo of producing poultry has gone up due to increased competition from imported Brazilian poultry. In November Chikondi is pressured to draw on any resources available to make his poultry profitable again for the Festive season in late December to cover the upcoming January school fees. As he is diagnosed with HIV/AIDS, he is receiving the antiretroviral (ARV) drug *efavirenz* free of charge from the hospital as part of a government programme. From a neighbour, he heard about poultry farmers giving part of their monthly ARV to the chickens, and how it increased their growth significantly.

Under the dire conditions, Chikondi decides to mix the ARV into the feed of the chickens resulting in quicker growth and bigger chickens to sell by the end of December. With the increased profits he is able to pay the school fees, but the decision turns out to have a number of impacts on human, animal and ecosystem health.

***Case vignette 2 workshop discussion, round 1***

- What happened in the case vignette?
- How did it happen?
- Why did it happen?
- How do you interpret the situation?
- Why and How is the case relevant to your health practice?

***Case vignette 2 continued***

Firstly, with the increased profits of quicker growth, Chikondi is inclined to continue sharing his prescribed ARVs with the chickens he raises. Over the coming months, this decision has severe implications for his own health as he is often getting sick and less able to take care of his poultry farm. With less energy and more sick days, the farm as a whole suffers, including the vegetables Chikondi grows for sustenance for his family. During a check-up at the hospital, tests show that the reduced ARVs in his body have led to the HIV rapidly multiplying, weakening his immune system. The doctors are also concerned that the less-than-inhibitory concentrations of ARVs in Chikondi´s body could also have long-term health consequences for AIDS patients in driving HIV resistance to the ARV *efavirenz*.

Secondly, regular customers of Chikondi´s chickens begin to complain that while larger the meat is less tasty and they experience it as less nutritious and they begin asking questions about what he has changed in the raising of the chicken and say that they much preferred the old chickens.

Thirdly, as part of safeguarding one of the major fishes contributing to Zimbabwean food security, government and NGOs are monitoring populations of tilapia fish in a nearby river finding liver damage and overall decline in the health of the fish. During a called meeting government representatives inform the community about the findings and how these initial test results could be linked to research coming out of South Africa on how pollution of the ARV efavirenz into aquatic environments like the nearby river is a health risk to *tilapia*. For the community, this is a major concern as many members rely on tilapia as a protein source in their diet and some wonder what other effects the ARVs used by the community members could have on the health of the river.

***Case vignette 2 workshop discussion, round 2***

*Additional questions for discussion*

- What ethical considerations emerge as part of the case?
- Are there alternative actions or interpretations of the situation?
- How could the situation be addressed?
